# Time-series clustering of cage-level sea lice data

**DOI:** 10.1371/journal.pone.0204319

**Published:** 2018-09-25

**Authors:** Ana Rita Marques, Henny Forde, Crawford W. Revie

**Affiliations:** 1 Centre for Veterinary and Epidemiological Research, Department of Health Management, Atlantic Veterinary College, University Prince Edward Island, Charlottetown, Prince Edward Island, Canada; 2 Måsøval Fiskeoppdrett AS, Sistranda, Norway; Scottish Association for Marine Science, UNITED KINGDOM

## Abstract

Sea lice *Lepeophtheirus salmonis* (Krøyer) are a major ectoparasite affecting farmed Atlantic salmon in most major salmon producing regions. Substantial resources are applied to sea lice control and the development of new technologies towards this end. Identifying and understanding how sea lice population patterns vary among cages on a salmon farm can be an important step in the design and analysis of any sea lice control strategy. Norway’s intense monitoring efforts have provided salmon farmers and researchers with a wealth of sea lice infestation data. A frequently registered parameter is the number of adult female sea lice per cage. These time-series data can be analysed descriptively, the similarity between time-series quantified, so that groups and patterns can be identified among cages, using clustering algorithms capable of handling such dynamic data. We apply such algorithms to investigate the pattern of female sea lice counts among cages for three Atlantic salmon farms in Norway. A series of strategies involving a combination of distance measures and prototypes were explored and cluster evaluation was performed using cluster validity indices. Repeated agreement on cluster membership for different combinations of distance and centroids was taken to be a strong indicator of clustering while the stability of these results reinforced this likelihood. Though drivers behind clustering are not thoroughly investigated here, it appeared that fish weight at time of stocking and other management practices were strongly related to cluster membership. In addition to these internally driven factors it is also possible that external sources of infestation may drive patterns of sea lice infestation in groups of cages; for example, those most proximal to an external source. This exploratory method proved useful as a pattern discovery tool for cages in salmon farms.

## Introduction

Sea lice *Lepeophtheirus salmonis* (Krøyer) is an important ectoparasite affecting farmed Atlantic salmon *Salmo salar* L. [1]. High infestation levels can be responsible for severe damage or even death to the host fish [2]. Combined treatment and management efforts typically lower sea lice abundances to levels that rarely impact fish health [3]. Although the parasite causes little direct mortality, it is responsible for high economic losses, making sea lice control a priority among salmon farmers and the scientific community, worldwide [4].

Since its emergence in the 1960s, Norwegian salmon farming has led the world in terms of the production of farmed salmon [5]. The impact of sea lice *L. salmonis* on Norwegian salmon farms was already apparent during the 1970s, and in 1997 the “National Action Plan Against Salmon Lice on Salmonids,” for regional and farm-level coordinated efforts, was implemented [6]. The Norwegian authorities have since developed and modified regulations and surveillance programs in an effort to control infestations in Norwegian salmon farms [7, 8]. Regulations include mandatory sea lice count reporting and a maximum threshold value, of 0.5 adult sea lice per fish, reduced to 0.2 adult female lice during the periods when most smolts are out-migrating [7, 9].

Apart from management and regulations, there is significant investment associated with the development and application of numerous treatment and prevention strategies at the farm level. Prevention and treatment strategies include the use of oral treatments in medicated feed, bath delousing through chemical treatments, and biological control using cleaner-fish [10]. New and innovative technologies have been applied to parasite management in salmon farming in Norway, such as ‘snorkel’ sea lice barrier technology [11] and cage enclosure in plankton sheeting [12].

Many factors can affect sea lice infestation levels, including the fact that cages may be stocked and harvested at different moments, treatments may be applied differentially between cages, and with varying effect, among other factors. Furthermore, events in one cage or group of cages can impact other cages differentially and in a way that cannot be measured with simple time-point comparisons, but which require a more flexible way of understanding sea-lice patterns of infection. This has led to the need for better analytical methods by which to understand how sea lice populations may vary synchronously within a farm to facilitate better management and evaluation of sea lice prevention and control efforts. This study makes use of time-series clustering strategies to discern common patterns in sea lice cage count data within three Norwegian farms. The general objective is to identify sets of cages that form homogenous groups, based on the sea lice counts, throughout a production cycle. Here we overcome the limitations of point-by-point clustering using time series clustering that takes into account the entire set of sea-lice time series counts for each cage. When such clustering is present, it may be that the commonalities in patterns is due to factors such as management practices or internal transmission between cages. It may also be the case that common sources of external infestation are impacting on sets of cages, causing the sea lice dynamics within these cages to follow similar patterns. Typically the types of clustering algorithm used here are designed as exploratory tools and do not allow for formal statistical inference regarding the causal mechanisms generating common patterns. However, in addition to their descriptive value they can generate hypotheses that can be explored using more traditional statistical methods.

## Materials and methods

### Data sets

The Norwegian sea lice surveillance protocol includes mandatory data collecting and reporting, which provides a wealth of surveillance time-series data of sea lice counts. The sea lice data sets used in the present study were supplied on request by 3 Norwegian farms, for a varying number of production cycles and within a time period from 2012 to 2017. The data consists of weekly, per-cage values of adult female lice, for varying numbers of sampled fish. The adult female lice counts are the most critical for overall sea lice population control, which aims to keep their values as low as possible. From January 2000, the limit for adult female lice per farm was set at 0.5 per fish, with values in excess of these levels requiring the application of control measures at the farm level within a two-week period [6]. Recently, the threshold value was lowered to 0.2 adult female lice per fish, during weeks 16 to 21 in the south of Norway, and weeks 21 to 26 in the north of Norway, specifically Nordland, Troms and Finnmark [9]. The data used in this study is provided in S1 File.

### Data preprocessing

Time-series clustering was performed by farm, considering the sea lice counts per cage as multivariate time-series objects, and for the entire sequence of time points that made up the salmon grow-out phase. Some cages started or completed their production at different times, meaning that some cages were considered to have no salmon and no sea lice during periods when other cages were producing fish. In order to consider time-series of equal lengths for all cages, these periods of non-production where given a sea lice count value of zero. All cage time-series data were equally spaced in time at one-week intervals and as such, any missing values were imputed using a linear Gaussian state-space model [13]. The final pre-processing step included a z-score normalization of the time-series data [14].

### Time-series clustering

Clustering is an unsupervised data mining technique by which homogenous groups, or clusters of objects, are formed with minimum inter-cluster and maximum intra-cluster similarity [15]. Time-series data are dynamic in the sense that they change over time, with time-series objects usually consisting of large numbers of observations (high dimensional data), making for interesting pattern discovery. Time-series clustering considers the complex nature of the data, aggregating large time-series objects into groups in what is a common exploratory technique in time-series visualization and comprehension [15].

Time-series clustering requires the definition of a clustering algorithm, a dissimilarity measure, a representative cluster centroid, and a cluster evaluation step [14]. It is not possible to know in advance what will be the best clustering approach for a given data set. To investigate the different partitions of cages and find which provide the best fit to the data, we apply several clustering algorithm configurations, for a total of 9 approaches, with cluster evaluation using 7 cluster evaluation indices, and a final step investigating cluster stability. In some cases, we expect clustering results to be consistent across different configurations, which may be an indication of clustering patterns in sea lice counts. However, given the unique behaviour of each algorithm and the inherent stochasticity in some of the methods, cluster membership would be expected to show variation between methods and even across random repetitions within the same method. Where cluster membership differs from total concordance within a specific method, stability is indicated to be ‘partial’ so as to contrast this with methods which result in largely identical membership and indicate highly stable clusters. However, it can be the case that two methods both result in stable clusters but that their membership differs between methods; this lack of stability is more problematic and indicates that treatment of one or more variables, or their use in the estimated distance metric is leading to inconsistent outcomes, making interpretation more challenging.

### Definition of the cluster algorithm

Time-series clustering algorithms are typically classified as data-based, working with the data as is and performing reduction methods that can be feature-based, extracting features for clustering; or, it can be model-based, extracting model parameters before clustering [15, 16]. Partitional clustering algorithms are raw-data-based and among the most explored and conventional clustering methods, such as the well-known k-means algorithm [15, 17]. Partitional clustering algorithms build “crispy” clusters, where each object belongs to a single cluster, and rely heavily on the choice of cluster centroid and the k chosen number of clusters [15]. For this reason, 3 centroid methods were investigated, as were all possible number of clusters, from 2 to k individual cage cluster members, where each cluster is a single cage.

### Definition of distance measure

Distance measures between time-series reflect their degree of similarity [14]. One of the most commonly used distance measures for time-series clustering is Euclidean distance [15]. Euclidean distance is parameter-free and frequently used as a benchmark for time-series dissimilarity searches [18–21]. Most other distance metrics are compared to the Euclidean distance metric, given its simplicity and stability [15]. In spite of being sensitive to noise, scale, and time shifts, Euclidean distance has been found to be very competitive [14].

To investigate whether patterns of sea lice counts vary among cages, where the effect of increased numbers of sea lice or treatment applications could yield a non-immediate effect on a neighbouring cage, we investigated similarity in time-series prioritizing the patterns of change over time. Dynamic Time Warping (DTW) is a distance measure that allows non-linear alignments between time-series to identify sequences similar in shape, even when misaligned and of different lengths [22, 23]. Events do not need to be perfectly aligned to be grouped by the algorithm, using DTW distance. In such a way, DTW allows for flexible sequential pattern discovery, adapted to real-world situations [24]. DTW was also chosen as a distance metric, overcoming many limitations associated with the use of Euclidean distance [25]. For faster computation and to prevent small sections of one time-series from mapping onto larger sections of another, a constraint, warping window is recommended [22]. A fixed window size of 9 weeks was selected for all datasets, to confer a biological basis for the choice in parameter, which would represent the 9 weeks for *Lepeophtheirus salmonis* generation time [26]. Note that a window size of zero is equivalent to applying the Euclidean distance measure [25]. Many authors believe that employing a correct warping window can increase classification accuracy [25, 27]. Ratanamahatana et al., (2004) investigated the effect of window size on classification accuracies and found that, for smaller data, the decrease in data size meant a decrease in classification accuracy, with peaks in accuracy appearing for increasingly larger window sizes. These authors also found that they were unable to simulate data sets that required more than a 10% window size to improve classification accuracy [25]. In this study, the window size of 9 weeks represents just less than 10% of the average length of the time-series data, for any of the production cycles analysed.

A third distance metric for time-series clustering was proposed by Cuturi et al., (2017) using Global Alignment Kernels (GAK) [28]. For GAK, similarities are based on kernels [14] that consider the cost over all possible alignment distances that map time-series onto each other as a positive kernel, which is more coherent than DTW alone [14, 28, 29].

### Definition of cluster centroid

Time-series centroids, also known as prototypes, can be considered as time-series that summarize the cluster around which they were built [14]. The most common centroid for clustering is the mean of each time point across the multiple time-series that make up the cluster [14]. A second common approach is to partition around medoids (PAM), the medoid being a representative time-series whose distance to all others in the cluster is minimal [14]. The third centroid function considered was developed by Petitjean et al. (2011): partitioning using DTW barycenter averaging (DBA) [30]. In this approach, representative time-series are used as centroids and the DTW alignments map to average values using the values within the cluster [14].

### Cluster evaluation

In the absence of labelled data, the internal cluster validity indices (CVIs) evaluate clustering results using information available in the data [15]. For each clustering method, the CVIs help determine the best k number of clusters [31]. There is no way of saying which CVI is best, so we simultaneously considered 7 CVIs. Briefly, the CVIs are based on the within-cluster cohesion and the between-cluster separation, relying on certain distances between time series points and centroids, and expressed in terms of the indices detailed in Table 1 [31].

**Table 1:** Clustering validity indices (CVI), the distances that are used as a measure of intra-cluster cohesion or inter-cluster separation and whether their value should be maximized or minimized when choosing the most robust set of clusters.

CVI	Cohesion	Separation	Goal
Calinski-Harabasz	Ponts to centroid	Cluster centroid to global centroid	Maximized
Davies-Bouldin and Davies Bouldin Modified	Points to centroid	Between centroids	Minimized
Silhouette	Between all points	Nearest-neighbor distance	Maximized
Dunn	Nearest-neighbor distance	Maximum cluster diameter	Maximized
COP	Points to centroid	Furthest neighbour distance	Minimized
Score function	Points to centroid	Cluster centroid to global centroid	Maximized

For the different number of k clusters considered per algorithm configuration, the CVI values were plotted and the steeper changes in values, either for maximization or minimization, helped indicate which was the best number of k partitions. Steeper drops or rises ending with improved CVI values would be preferred. Additionally, if the CVI value is followed by a steep change that indicates worse performance, then the value it drops/increases from is chosen. If the CVIs disagreed on the number of clusters that result in the best partitioning, the majority vote was used to choose the best k cluster value. Furthermore, if consecutive CVI values were steadily and increasingly better, the smallest k was chosen, since it was expected that the CVIs perform better on increasingly smaller clusters.

Due to their random choice of initial cluster members, partitional clustering procedures are stochastic in nature [14]. The best clustering algorithm configuration can also be evaluated through its stability, without the assumption of compactness, by quantifying the agreement between 20 and 100 random repetitions of the clustering algorithm [14, 31]. With the help of the clue package, the clusters can be evaluated against the medoid of the random repetitions of the same clustering algorithm through a confusion matrix to identify all, partial, or no correctly predicted cluster memberships [32].

### Statistical software

The time-series clustering algorithms were implemented in R statistical computing software and the majority of the time-series analyses were performed using the R package dtwclust [33, 34]İmputation of missing values was performed using the imputeTS package, and clustering stability was performed using the source code in dtwclust and the R clue package [32, 35].

## Results

There was no single best CVI to determine the ‘ideal’ number of clusters across all data sets. For the majority of cases, the Score Function index was relatively constant and for this reason was eliminated from the analysis. There was a tendency for CVIs to show an improvement for the maximum value of k, which is a natural consequence of the lack of intra-variance and high inter-variance, given single-element clusters.

**Fig 1:**
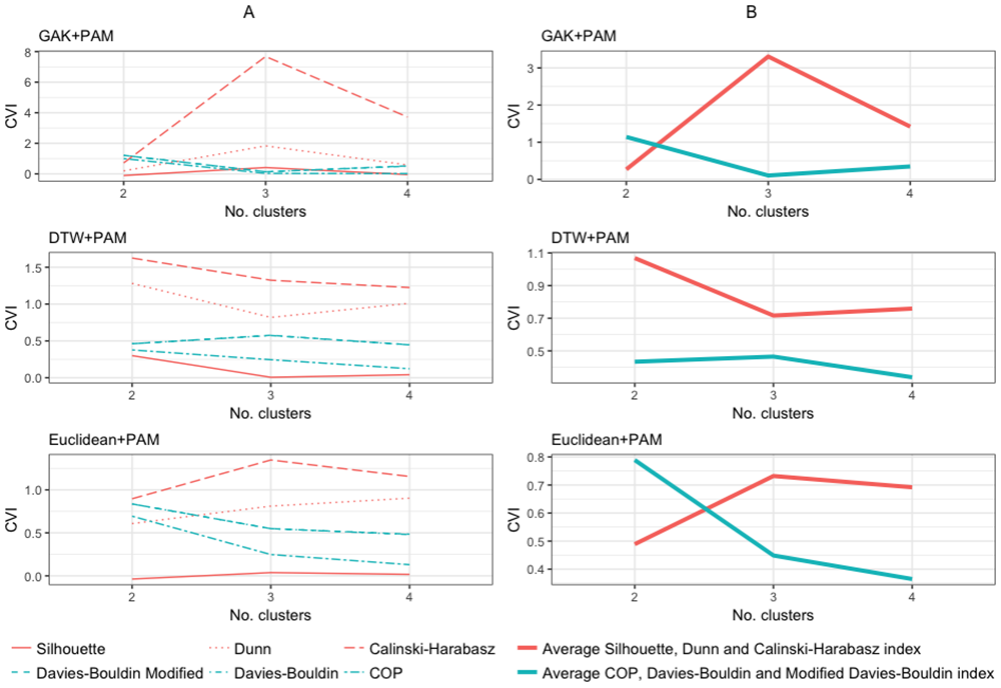
Farm I CVI plots. Cluster validity index plots for the most stable algorithms applied to the time-series data for Farm I in 2012-2013. (A) individual CVI plots; (B) average values for each group of CVIs.

For the 2012-2013 time-series for Farm I, the CVI plots for 3 of the most stable algorithms are shown in Fig 1. The average values for the CVIs, according to whether these belonged to the group of CVIs for which decreased or increased values indicate an improvement, are shown as blue and red lines, respectively (Fig 1). In most cases, the Calinski-Harabasz index dominated the behaviour of the red line, while the Davies-Bouldin and Davies-Bouldin modified indexes, which sfrequently overlap, tended to drive changes in the blue line. In some instances, CVIs behaved in agreement as the number of clusters changed, as was the case in GAK+PAM 2012-2013 and the choice of k = 3. However, consensus between CVIs was not always apparent. Examples of such behaviour can be seen for the DTW+PAM and the Euclidean+PAM based clusters for 2012-2013. For the DTW+PAM algorithm, two clusters were determined as the best k, where CVIs worsened with easily identified steep changes, from 2 to 3 clusters, with the exception of the COP index. Thereafter, most CVI values improved when moving from 3 to 4 clusters, with the exception of the Calinski-Harabasz index. The choice of 2 clusters rather than 4 relied on the fact that the steepest change in values occurred for the change from 2 to 3 clusters, and was reinforced by the fact that three CVI values at k = 4 had similar or worse values than those seen at k = 2. An increased number of clusters will typically lead to apparent improvement in the performance based on CVI values, as there is a natural tendency for the variance within groups to decrease and increase the variance between clusters. This is analogous to over-fitting in the modelling context.

The Euclidean+PAM algorithm for the same production cycle (2012-2013) revealed a fairly steady improvement for the CVIs that should be minimized to indicate better clustering, as well as for the indices to be maximized, with the exception of the Calinski-Harabasz index. The choice of 3 clusters was clearly supported by the Calinski-Harabasz index with the inflection in value from k = 3 to k = 4. For the Dunn index a steeper improvement occurred for the change from 2 to 3 clusters, and also improved from 3 to 4 which, as previously mentioned, is to be expected and should not be over-interpreted.

**Fig 2:**
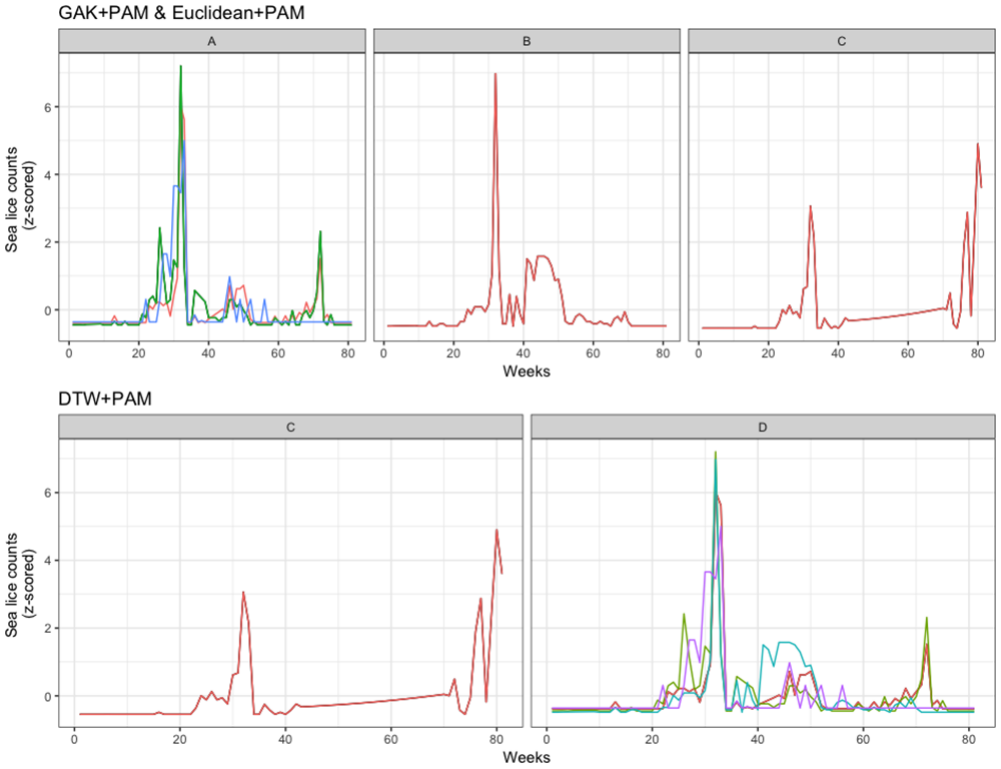
Farm I clustering for 2012-2013. Time-series plots for the most stable clustering algorithms for Farm I, production cycle of 2012-2013.

**Fig 3:**
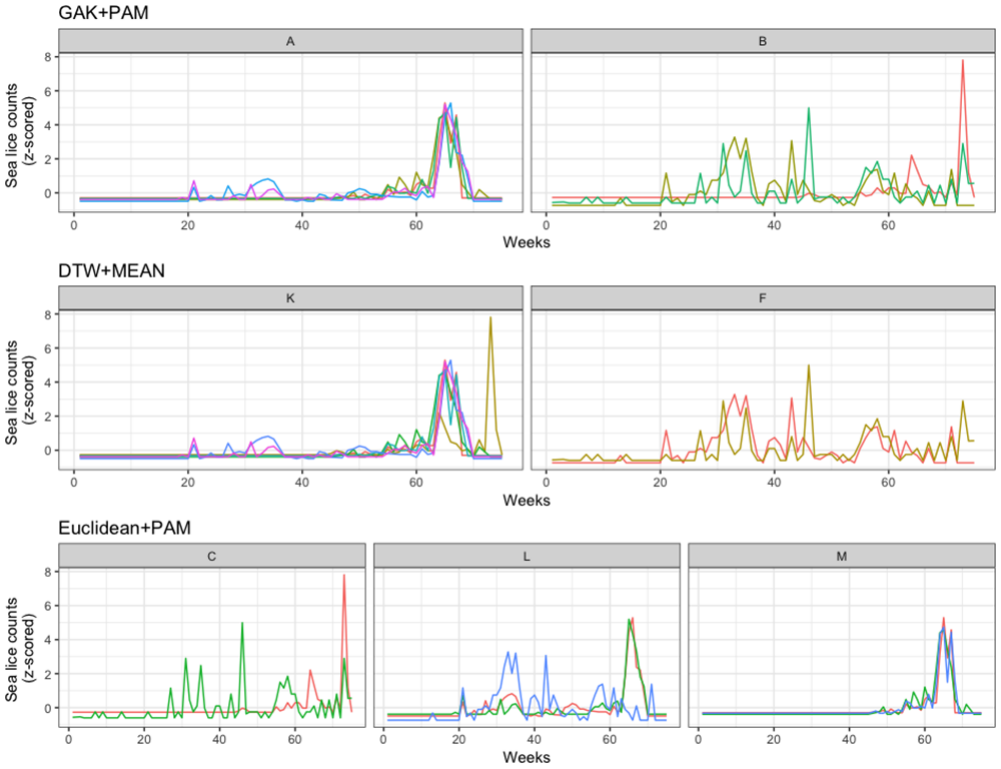
Farm I clustering for 2014-2015. Time-series plots for the most stable clustering algorithms for Farm I, production cycle of 2014-2015.

**Fig 4:**
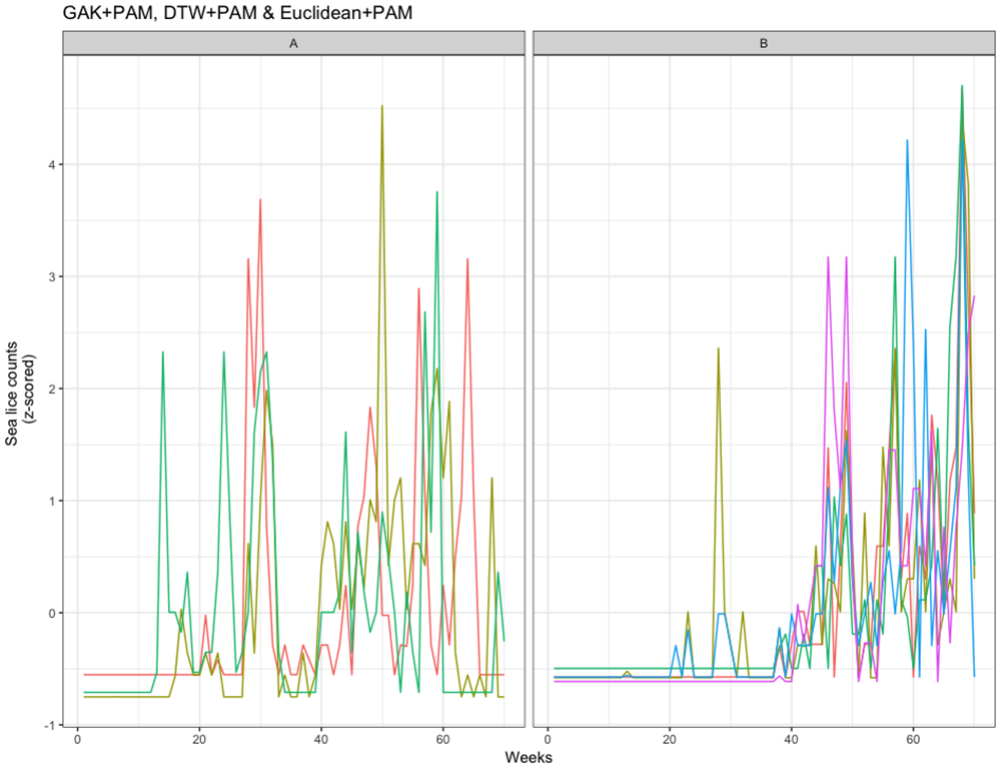
Farm I clustering for 2016-2017. Time-series plots for the most stable clustering algorithm for Farm I, production cycle of 2016-2017.

The clustering results are presented for Farm I in Table 2 for each of the three production cycles, with the most stable clustering results shown in Figs 2, 3 and 4. For the 2012 to 2013 production cycle, the clustering algorithms identified 2 or 3 clusters. In almost all cases cages 2, 3, and 5 were in a cluster, sometimes joined by cage 4, while cage 1 was always a “singleton” cluster (Fig 2). Furthermore, the GAK algorithms and Euclidean algorithms produced the same cluster memberships, suggesting a strong clustering pattern for 2012-2013.

**Table 2:** Clustering results for Farm I, with cluster membership as shown in the plots in Figs 2, 3 and 4. The most stable clusters are identified per cycle, alphabetically.

Production cycle from 2012-03-23 to 2013-10-03				
Distance metric	Centroid	No. clusters	Cluster members	Stability 20;100*
GAK	PAM	3	A{2,3,5};B{4};C{1}	all;all
	DBA	3	A{2,3,5};B{4};C{1}	all;all
	MEAN	3	A{2,3,5};B{4};C{1}	all;all
DTW	PAM	2	C{1};D{2,3,4,5}	all;all
	DBA	3	C{1};E{3};F{2,4,5}	nn;nn
	MEAN	2	C{1};D{2,3,4,5}	all;all
Euclidean	PAM	3	A{2,3,5};B{4};C{1}	all;ptt
	DBA	3	A{2,3,5};B{4};C{1}	all;ptt
	MEAN	3	A{2,3,5};B{4};C{1}	all;ptt
Production cycle from 2014-05-18 to 2015-10-17				
Distance metric	Centroid	No. clusters	Cluster members	Stability 20;100*
GAK	PAM	2	A{1,3,4,5,6};B{2,7,8}	all;all
	DBA	2	A{1,3,4,5,6};B{2,7,8}	all;all
	MEAN	3	A{1,3,4,5,6};C{2,8};D{7}	all;all
DTW	PAM	4	E{4};F{7,8};G{1,2};H{3,5,6}	nn;nn
	DBA	2	I{1,2,3,4};J{5,6,7,8}	ptt;ptt
	MEAN	2	F{7,8};K{1,2,3,4,5,6}	all;all
Euclidean	PAM	3	C{2,8};L{5,6,7};M{1,3,4}	all;all
	DBA	2	M{1,3,4};N{2,5,6,7,8}	ptt;ptt
	MEAN	3	A{1,3,4,5,6};F{7,8};O{2}	ptt;all
Production cycle from 2016-03-03 to 2017-06-29				
Distance metric	Centroid	No. clusters	Cluster members	Stability 20;100*
GAK	PAM	2	A{4,7,8};B{1,2,3,5,6}	all;all
	DBA	2	A{4,7,8};B{1,2,3,5,6}	all;all
	MEAN	2	A{4,7,8};B{1,2,3,5,6}	all;all
DTW	PAM	2	A{4,7,8};B{1,2,3,5,6}	all;all
	DBA	2	A{4,7,8};B{1,2,3,5,6}	all;all
	MEAN	2	A{4,7,8};B{1,2,3,5,6}	all;all
Euclidean	PAM	2	A{4,7,8};B{1,2,3,5,6}	all;all
	DBA	2	C{4};D{1,2,3,5,6,7,8}	ptt;ptt
	MEAN	2	E{4,8};F{1,2,3,5,6,7}	ptt;ptt

*ptt: partial; nn: none; GAK: Global Alignemnt Kernels; DTW: Dynamic Time Warping; PAM: Partition Around Medoids; DBA: Dynamic Barycenter Averaging.

In the production cycle of 2014 to 2015, an additional 3 cages were present, giving a total of 8 cages. This cycle was characterized by a less obvious partitioning pattern, with cluster results varying, depending on the distance metric applied (see Table 2 and Fig 3). All but two algorithms supported clustering of cages 7 and 8, and all but three supported the clustering of cages 3, 5, and 6. As was the case for the first production cycle, the GAK distance metric provided the most stable clustering results, for both 20 and 100 randomizations.

During the third production cycle, 2016 to 2017, the clustering results were fairly consistent within and between algorithms (Table 2 and Fig 4). The clustering algorithms identified two large clusters of possible cage-level related sea lice behaviour. Most of the algorithms supported clustering of cages 7 and 8, as was the case of the previous production cycle, though in this cycle they also almost always clustered with cage 4. Likewise, cages 3, 5, and 6 often clustered together during both production cycles, though in this latter cycle they were always clustered with cages 1 and 2. Furthermore, the two algorithms that did not identify identical cluster membership of A and B (Euclidean+DBA and Euclidean+MEAN), also failed to produce repeatable results for both 20 and 100 randomizations. Overall, the similarity of cluster memberships suggests two similar groups of sea lice populations over the production cycle of 2016 to 2017.

**Fig 5:**
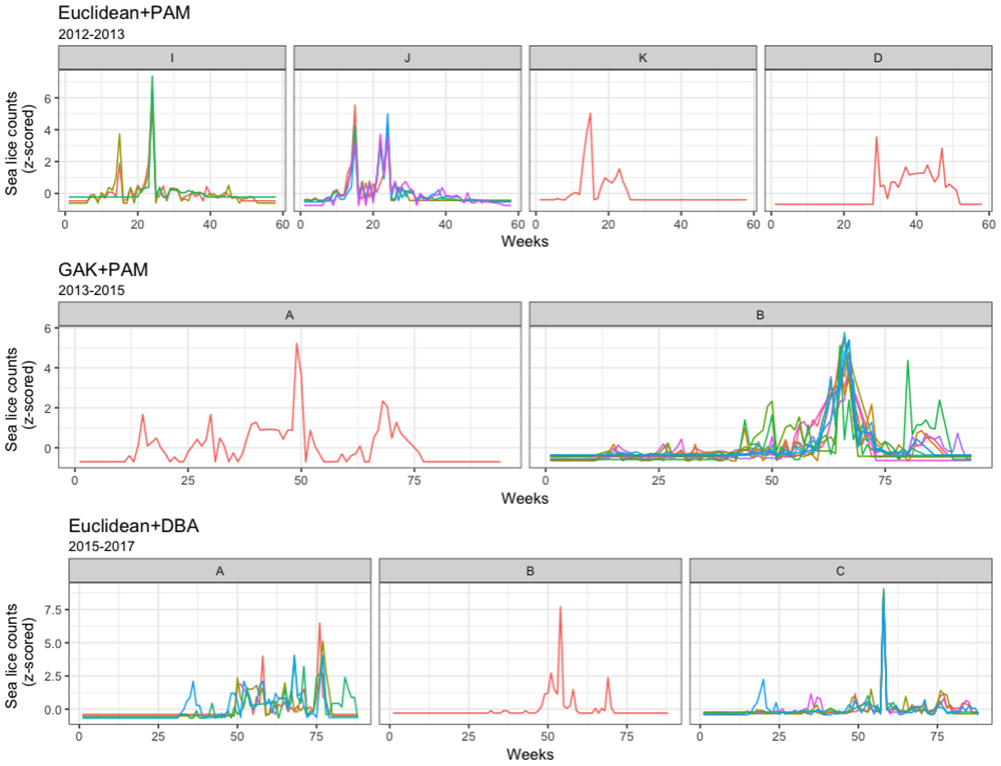
Farm II clustering per production cycle. Time series plots for the most stable clustering algorithm for Farm II, one from each production cycle.

Clustering based on sea lice data from Farm II’s production cycle in 2012-2013 is summarized in Table 3, and a stable configuration is shown in Fig 5. The CVIs for GAK distance metric with PAM or DBA, suggested 4 clusters but with unstable results. When investigating the stability of 2 clusters with such algorithms, clusters were also unstable. However, if the centroid is the simple average of time-series counts, most algorithms identify one cluster with a single member (cage 10), and with stable results. This singleton cluster is also identified in the DTW and Euclidean algorithms. For the farm’s second production cycle (2013-2015), the algorithms produced similar results, identifying two clusters: a small cluster consisting of cage 6 and/or cage 4, with the second cluster consisting of all remaining cages. The low value of k and the unstable results for 2013-2015 suggest an absence of clustering and a similarity of sea lice count patterns among cages (Fig 5). During the final production year (2015-2017, Table 3), similar cluster membership was found among the algorithms, with clustering of cages 2, 6, 7, 9, and 10, of cages 4 and 5 (often with cages 1 and/or 3), and with cage 8 mostly identified as a singleton cluster. Overall, the agreement among cluster memberships at Farm II would suggest that over the final two production cycles the sea lice count patterns were similar among all cages, with the exception of a few singleton cage clusters. The lack of agreement for the first production cycle of 2012-2013 deserves comment. Here, the two algorithm configurations that produced repeatable results, GAK+MEAN and Euclidean+PAM, identified both a large cluster and smaller clusters with fewer (often only one) member(s).

**Table 3:** Clustering results for Farm II. The most stable clusters are identified per cycle, alphabetically.

Production cycle from 2012-04-20 to 2013-05-25				
Distance metric	Centroid	No. clusters	Cluster members	Stability 20;100*
GAK	PAM	4	A{5};B{3,4,8,9};C{1,2,6,7};D{10}	ptt;ptt
	DBA	4	A{5};B{3,4,8,9};C{1,2,6,7};D{10}	ptt;ptt
	MEAN	2	D{10};E{1,2,3,4,5,6,7,8,9}	all;all
DTW	PAM	4	D{10};F{3,5};G{4,7,8,9};H{1,2,6}	ptt;ptt
	DBA	4	D{10};F{3,5};G{4,7,8,9};H{1,2,6}	ptt;ptt
	MEAN	4	D{10};F{3,5};G{4,7,8,9};H{1,2,6}	all;ptt
Euclidean	PAM	4	D{10};I{3,4,5};J{2,6,7,8,9};K{1}	all;all
	DBA	4	D{10};F{3,5};L{6,7,8,9};M{1,2,4}	ptt;ptt
	MEAN	4	D{10};F{3,5};L{6,7,8,9};M{1,2,4}	ptt;ptt
Production cycle from 2013-08-30 to 2015-06-5				
Distance metric	Centroid	No. clusters	Cluster members	Stability 20;100*
GAK	PAM	2	A{4};B{1,2,3,5,6,7,8,9,10,11,12}	ptt;all
	DBA	2	C{4,5,6};D{1,2,3,7,8,9,10,11,12}	ptt;ptt
	MEAN	2	E{4,6};F{1,2,3,5,7,8,9,10,11,12}	ptt;ptt
DTW	PAM	2	G{6};H{1,2,3,4,5,7,8,9,10,11,12}	ptt;ptt
	DBA	2	G{6};H{1,2,3,4,5,7,8,9,10,11,12}	ptt;ptt
	MEAN	2	G{6};H{1,2,3,4,5,7,8,9,10,11,12}	ptt;ptt
Euclidean	PAM	2	G{6};H{1,3,4,5,6,7,8,9,10,11,12}	ptt;ptt
	DBA	2	G{6};H{1,2,3,4,5,7,8,9,10,11,12}	all;ptt
	MEAN	3	A{4};F{1,2,3,5,7,8,9,10,11,12};G{6}	ptt;all
Production cycle from 2015-08-09 to 2017-04-07				
Distance metric	Centroid	No. clusters	Cluster members	Stability 20;100*
GAK	PAM	3	A{1,3,4,5};B{8};C{2,6,7,9,10	ptt;all
	DBA	3	A{1,3,4,5};B{8};C{2,6,7,9,10}	nn;all
	MEAN	4	B{8};C{2,6,7,9,10};D{1,3,5};E{4}	all;ptt
DTW	PAM	3	F{4,5};G{1,3};H{2,6,7,8,9,10}	all;all
	DBA	2	I{3,4,5};J{1,2,6,7,8,9,10}	ptt;ptt
	MEAN	2	I{3,4,5};J{1,2,6,7,8,9,10}	ptt;ptt
Euclidean	PAM	2	B{8};K{1,2,3,4,5,6,7,9,10}	ptt;ptt
	DBA	3	A{1,3,4,5};B{8};C{2,6,7,9,10}	all;all
	MEAN	3	B{8};I{3,4,5};L{1,2,6,7,9,10}	all;ptt

*ptt: partial; nn: none; GAK: Global Alignemnt Kernels; DTW: Dynamic Time Warping; PAM: Partition Around Medoids; DBA: Dynamic Barycenter Averaging.

**Fig 6:**
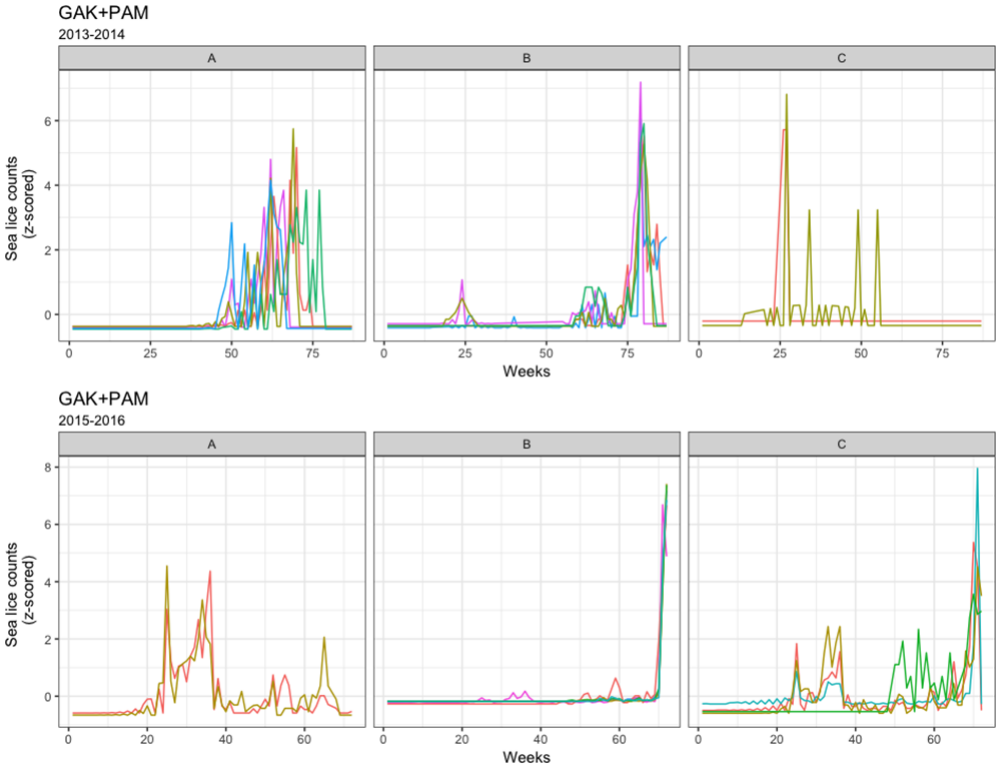
Farm III clustering per production cycle. Time series plots for the most stable clustering algorithm for Farm III, one from each production cycle.

The various clustering algorithms produced consistent results for the third farm, Farm III (Table 4). For the first production cycle, all 9 algorithms agreed on cage membership of the three clusters identified, shown in the upper panel of Fig 6. For the second production cycle of 2015-2016, the clustering algorithms produced similar results, suggesting the existence of 2 or 3 clusters, and the frequent clustering of cages 4 and 6, and of cages 2, 3, 5, 9, 11, and 12. The cluster membership of cages was not shared between the two production cycles. Overall, the agreement between algorithms suggests strong clustering at Farm III, with a large number of cages having a similar sea lice infestation pattern, while a few cages exhibit a different pattern in both production cycles.

**Table 4:** Clustering results for Farm III. The most stable clusters are identified per cycle, alphabetically.

Production cycle from 2013-03-24 to 2014-11-13				
Distance metric	Centroid	No. clusters	Cluster members	Stability 20;100*
GAK	PAM	3	A{3,5,6,7,11};B{1,4,9,10,12};C{2,8}	all;all
	DBA	3	A{3,5,6,7,11};B{1,4,9,10,12};C{2,8}	all;all
	MEAN	3	A{3,5,6,7,11};B{1,4,9,10,12};C{2,8}	all;all
DTW	PAM	3	A{3,5,6,7,11};B{1,4,9,10,12};C{2,8}	all;all
	DBA	3	A{3,5,6,7,11};B{1,4,9,10,12};C{2,8}	all;all
	MEAN	3	A{3,5,6,7,11};B{1,4,9,10,12};C{2,8}	ptt;ptt
Euclidean	PAM	3	A{3,5,6,7,11};B{1,4,9,10,12};C{2,8}	all;all
	DBA	3	A{3,5,6,7,11};B{1,4,9,10,12};C{2,8}	all;all
	MEAN	3	A{3,5,6,7,11};B{1,4,9,10,12};C{2,8}	all;all
Production cycle from 2015-05-04 to 2016-09-05				
Distance metric	Centroid	No. clusters	Cluster members	Stability 20;100*
GAK	PAM	3	A{4,6};B{2,3,5,9,11,12};C{1,7,8,10}	all;all
	DBA	2	A{4,6};D{1,2,3,5,7,8,9,10,11,12}	all;all
	MEAN	2	A{4,6};D{1,2,3,5,7,8,9,10,11,12}	all;all
DTW	PAM	2	E{1,4,6,7};F{2,3,5,8,9,10,11,12}	ptt;ptt
	DBA	2	E{1,4,6,7};F{2,3,5,8,9,10,11,12}	ptt;ptt
	MEAN	2	G{1,4,6,7,8};H{2,3,5,9,10,11,12}	all;all
Euclidean	PAM	3	A{4,6};B{2,3,5,9,11,12};C{1,7,8,10}	all;all
	DBA	2	A{4,6};D{1,2,3,5,7,8,9,10,11,12}	all;all
	MEAN	4	A{4,6};B{2,3,5,9,11,12};I{8};J{1,7,10}	all;all

*ptt: partial; nn: none; GAK: Global Alignemnt Kernels; DTW: Dynamic Time Warping; PAM: Partition Around Medoids; DBA: Dynamic Barycenter Averaging.

## Discussion

The clustering algorithms explored frequently produced similar and stable results. The GAK algorithms provided the most stable results across farms and production cycles. The flexibility of DTW, compared to the results of the less plastic Euclidean distance metrics, was not apparent when evaluating cluster stability: it may be that the nature of the data does not require such flexibility. Additionally, clusters were frequently made up of a large number of members, contradicting the sometimes posited “worst cage” phenomenon [36].

Possible drivers behind the clustering include time of stocking and harvesting, as well as the sorting and splitting of salmon between cages. Another possible driver is fish weight, frequently an indicator of life stage and development. To informally explore these factors, we reviewed management practices at Farm III, where the clustering algorithms produced consistent results. For the time series from 2013 to 2014, clustering of cages 2 and 8 was frequent among algorithms. These cages were stocked at the same time and with fish of similar weights. They were also depopulated at the same time. The remaining cages were stocked and harvested at very different times and the fish in those cages were harvested at significantly higher weights. Weight and time of harvest was also considered a driving force behind sea lice abundance for cages 3, 5, 7 and 11. These cages started production half way through the cycle and were stocked with larger fish from other cages, while cages 1 and 9, the remaining late starters, were stocked with lower weight individuals compared to neighbouring cages. The following cycle at Farm III, for 2015 to 2016, included clustering of cages 4 and 6, two cages subject to sorting and splitting. Here the largest fish were selected and kept in their cage, raising the weight at harvest in these two cages. Examples suggesting weight and management as driving forces behind sea lice clustering also occurred at Farms I and II. Further research into the potential for management factors to influence clustering between cages should be investigated.

Due to the nature of sea lice count data, any clustering must also consider the similarity in sea lice populations among clustered cages in the context of their varying response patterns to treatment interventions. Due to inherent limitations, the algorithms were unable to identify a global cluster, consisting of all the farm cages. It was also challenging to determine at which point each cage may be forming its own individual cluster, as might be expected if treatment effects and transmission were largely random. Either of these two scenarios would most likely occur where unstable clustering and discordant cluster membership among the algorithms was frequent. In the analysis presented here the algorithm was applied to all records within each production cycle: the usefulness of this approach as a monitoring tool, when applied to smaller datasets of fewer records, could also be explored.

It may also be the case that such clustering analyses could clarify the relative importance of internal versus external sources of infestation. For example, it may be expected that if the primary mechanism was internal re-infestation, then all cages would show similar patterns and thus form a single or at least large clusters. Conversely, if infestation was primarily from external sources then it might be expected that certain cages, closer to the location from which such infestation was arriving would be infected earlier and/or more heavily than other cages. It may also be the case that infestation due to Caligus species, which are known to present a different infestation profile on salmon farms, could be more clearly identified using clustering analyses [37].

## Conclusion

Clustering of disease is a well-known concept in aquaculture: clustering has been explored for identification of spatial-temporal transmission of disease, and explored at the cage level, to develop improved sampling protocols for sea lice sampling [38–41]. As an unsupervised learning tool, clustering has been used to characterize aquaculture production systems [42, 43]. Here we extended clustering of time-series data for pattern discovery outside of the habitual biological field of application for gene expression data, for clustering cage-level sea lice count data [15]. To our knowledge, this is the first application of such time-series clustering approaches to identify common parasite population signatures in the context of aquaculture. The exploratory analysis of the time series cage-level data for adult female sea lice abundance allowed us to identify clustering patterns across cages in three Norwegian farms; within a given production cycle and, in some cases, among production cycles. We focused on three algorithm configurations that allowed us to explore the patterns in varying ways, but which, in most instances, produced generally consistent results. Time-series clustering is useful for understanding cage-to-cage sea lice population dynamics and interpreting the effect of management strategies at the farm level. It is also likely that these approaches could be used to explore broader patterns of sea lice infestation among a large number of farms, and thus identify clusters which exhibit similar dynamics as an initial step to exploring the causal mechanisms that may underlie such commonalities. The results demonstrate the potential of time-series clustering algorithms for pattern discovery in aquaculture.

## Supporting information

S1 File Cage-level data. The cage-level data file for the 3 farms in the analysis.
(CSV)
